# Urinary Metabolomics in Young Soccer Players after Winter Training Season

**DOI:** 10.3390/metabo12121283

**Published:** 2022-12-17

**Authors:** Hyang-Yeon Kim, Jung-Dae Lee, Yun-Hwan Lee, Sang-Won Seo, Ho-Seong Lee, Suhkmann Kim, Kyu-Bong Kim

**Affiliations:** 1 College of Pharmacy, Dankook University, 119 Dandae-ro, Cheonan 31116, Republic of Korea; 2 Center for Human Risk Assessment, Dankook University, 119 Dandae-ro, Cheonan 31116, Republic of Korea; 3 Department of Exercise and Medical Science, Graduate School, Dankook University, 119 Dandae-ro, Cheonan 31116, Republic of Korea; 4 Department of Sports Science, Gwangju University, Gwangju 61743, Republic of Korea; 5 Department of Chemistry and Chemistry Institute for Functional Materials, Pusan National University, Busan Daehak-ro 63 beon-gil 2, Busan 46241, Republic of Korea

**Keywords:** winter training season, young soccer players, NMR analysis, multivariate analysis, recovery

## Abstract

During the off-season, soccer players in Korea attend the winter training season (WTS) to build running stamina for the next season. For young soccer players, proper recovery time is needed to prevent injury or muscle damage. In this study, urinary metabolites in young players after 1, 5, and 10 days of the WTS were analyzed using nuclear magnetic resonance spectroscopy (NMR) combined with multivariate analysis to suggest appropriate recovery times for improving their soccer skills. After NMR analysis of the urine samples obtained from young players, 79 metabolites were identified, and each group (1, 5, or 10 days after WTS) was separated from the before the WTS group in the target profiling analysis using partial least squares-discriminant analysis (PLS-DA). Of these, 15 metabolites, including 1-methylnicotinamide, 3-indoxylsulfate, galactarate, glutamate, glycerol, histamine, methylmalonate, maltose, *N*-phenylacetylglycine, trimethylamine, urea, 2-hydroxybutyrate, adenine, alanine, and lactate, were significantly different than those from before the WTS and were mainly involved in the urea, purine nucleotide, and glucose-alanine cycles. In this study, most selected metabolites increased 1 day after the WTS and then returned to normal levels. However, 4 metabolites, adenine, 2-hydroxybutyrate, alanine, and lactate, increased during the 5 days of recovery time following the WTS. Based on excess ammonia, adenine, and lactate levels in the urine, at least 5 days of recovery time can be considered appropriate.

## 1. Introduction

The game of soccer is a physical activity that requires sprinting, intercepting a pass, dribbling, jumping, passing the ball to a teammate, and shooting for 90 min, amounting to approximately 723 ± 203 actions [1]. Therefore, the aerobic ability to generate energy and anaerobic power for agility and sprinting off the ball are the most important physical fitness factors [2]. During the season, players need a lot of stamina and experience injuries. After the season, players usually take a short rest and perform light exercises to prevent muscle damage and maintain their skills. Before the next season opens, players attend the winter training season (WTS) to build running stamina for the next season. During the WTS, players are trained in cardio exercises, such as running, jumping, showjumping, sprinting, and weight exercises using dumbbells and barbells [3]. After attending the WTS, basic physical fitness and total exercise time were significantly increased, indicating an improvement in the participants’ soccer play sense and physical skills at each position [3]. Therefore, participating in the WTS is vital for maintaining fundamental physical strength and improving soccer skills for soccer players. However, in the case of young soccer players, highly intense physical training like that undertaken by adult players is not required because they have not fully developed and have a high risk of injury. Moreover, the ages between 12 and 15 years are associated with increased growth hormone and muscle mass [4]. Therefore, young players are advised not to put their bodies under too much pressure but are trained instead using profitable exercises for no longer than two hours. In addition, sufficient rest time between each training session allows the participants to recover physically. Recovery from exercise is physiologically needed for additional training, and, based on the cardiovascular system, hemodynamic parameters, such as arterial pressure and conductance of systemic vascular and skeletal muscles, were observed to change after exercise [5,6,7]. However, proper recovery times (hours or days) after finishing the WTS to repair and strengthen the body have not been reported for young players.

Metabolite profiling identifies the meaningful metabolites among numerous metabolites to understand the role of small metabolites in disease, classification, and diagnostics [8,9,10,11]. To effectively handle and process a large amount of information, statistical analysis methods, such as multivariate analysis, are performed, in which multiple measurements are set on variables and significant interrelationships among the variables are evaluated [12]. Nuclear magnetic resonance spectroscopy (NMR) was used for the urinary metabolite analysis in this study, a robust technique for detecting, identifying, and quantitating compounds. Urine contains various metabolites eliminated through the kidneys, which are filtered by the plasma glomeruli, excreted by the renal tubules, and secreted by the urogenital system. A urine sample can be obtained easily and is used to determine whether banned substances, such as hormones or drugs, have been consumed. For example, steroids, such as testosterone, androsterone, etiocholanolone, 5α-androstan-3α, 17β-diol, and 5β-androstan-3α, can be detected by gas chromatography–mass spectrometry (GC–MS) analysis, and the ratios of these steroids in urine can act as biomarkers for the doping test [13]. It is also used as a diagnostic marker for diabetes, hypertension, and hyperlipidemia [14,15]. In addition, it is considered excellent for reflecting the condition of athletes in various sports without causing harm. Therefore, many studies have recently been conducted using metabolomics to monitor the stamina of players and to properly design training programs [16]. Vike et al. have reported that some metabolites in urine, such as xanthine, fatty acids, and primary bile acids, could be used as biomarkers for pre- to post-season changes in soccer athletes [17]. In addition, Kim et al. and Jang et al. reported that when athletes suffered from skeletal muscle damage, the levels of creatine kinase and myoglobin [18], as well as those of amino acids, organic acids, and sugars [19], were altered.

As mentioned above, no high-intensity exercise for young players was performed in the WTS, and recovery time was provided after the WTS. However, the recovery time needed for young players to achieve better conditioning has not been reported. Therefore, in this study, urinary metabolites obtained from the young players after finishing the WTS were analyzed by NMR combined with multivariate analysis to suggest proper recovery times for improving their soccer skills.

## 2. Materials and Methods

Subjects. Fourteen male subjects (10–13 years; weight: 35.86 ± 4.37 kg, height: 143.17 ± 4.39 cm, muscle: 15.84 ± 1.79 kg, body fat: 15.73 ± 5.387 kg, and body mass index: 17.52 ± 2.08, presented as mean ± standard deviation,) were included in the study. Weight, height, muscle mass, body fat, and body mass were measured using the body composition analyzer (Inbody 770, Biospace, Seoul, Korea). Individual information is presented in Table S1. All participants had more than 1 year of soccer experience and had been participating in the club for over 2 h a day 5 or more times a week. This study was approved by the Ethics Committee of Dankook University in accordance with the ethical standards of the Declaration of Helsinki (DKU 2020-07-004-001).

Winter training season (WTS). In this study, the subjects performed morning and afternoon training 5 days a week. The exercise protocol followed that described by Gamble et al. and Stenger et al. [20,21] and is presented in Figure S2.

Urine collection. Urine samples were collected before WTS (Bef), and 1 (Aft), 5 (Aft-5D), and 10 days (Aft-10D) after the WTS (Figure S1). All samples were taken at 10:00 a.m. on an empty stomach and stored at −70 °C until analysis.

^1^H NMR spectroscopic analysis. To evaluate the metabolic changes in urine before and after WTS, urine samples were thawed to 4 °C and centrifuged for 5 min at 900× *g* to remove solids. A 600 μL aliquot of the supernatant was added to a microcentrifuge tube containing 70 μL of D_2_O solution with 5 mM DSS and 100 mM imidazole. DSS was used as the internal standard reference for the chemical shift scale, and imidazole was used for ^1^H-NMR analysis to obtain more proton signals. In addition, 30 μL of 0.42% sodium azide was added to avoid bacterial reproduction during NMR analysis. After vortexing, the solution pH was adjusted to 6.8, and the urine sample was analyzed using a Varian Unity Inova 600 MHz NMR spectrometer within 48 h at the Pusan National University. One-dimensional NMR spectra were acquired at 26 °C with the following acquisition parameters: spectral width of 24,038.5 Hz, 12.53 min acquisition time, and 128 nt. Additionally, a relaxation delay of 3 s and saturation power of 4 was set to suppress massive water peaks. The acquired NMR spectra were phased, baseline-corrected, and referenced to the DSS peak (chemical shift of 0 ppm) using VnmrJ 4.2 software (Agilent Technologies, Santa Clara, CA, USA). The spectral region corresponding to water (δ4.5–5.0) was removed from the analysis to prevent variation in water suppression efficiency. Identification (pattern analysis) and quantification of the NMR spectra were performed using the Chenomx NMR Suit program (ver. 4.6; Chenomx Inc., Edmonton, AB, Canada) with the Chenomx library database. In this study, signals that overlapped or were too small for intensity calculation were not considered for accurate quantitation. Metabolite concentrations were expressed as relative ratios normalized to creatinine concentration, assuming a constant rate of creatinine excretion in each urine sample.

Multivariate analysis. All data were converted from the NMR software format into Microsoft Excel format (Microsoft, Seattle, WA, USA). One-dimensional NMR spectral data were imported into SIMCA-P (version 12.0; Umetrics Inc., Kinnelon, NJ, USA) for multivariate statistical analysis to examine intrinsic variations in the data set. The data were scaled using unit variance (UV) scaling prior to principal component analysis (PCA) and partial least squares-discriminant analysis (PLS-DA). PCA and PLS-DA score plots were used to interpret intrinsic variations in the data. In addition, PLS-DA between before and 1 (or 5, or 10) days after WTS was conducted, and VIP values from each group were presented in Figures and Tables. The permutation test was conducted with 20 randomly initiated permutations in a PLS-DA model. Pathway and enrichment analysis of selected metabolites by clustering in PLS-DA was performed using MetaboAnalyst 5.0 [22].

Statistical analysis. All data are presented as mean ± SD. Statistical calculations were performed using Excel 2013 (Microsoft for Windows) and GraphPad Prism Software version 5.04 (San Diego, CA, USA). Statistical analysis was conducted using GraphPad Prism software to compare the levels of each metabolite, and *p* < 0.05 was considered statistically significant. Changes in urinary metabolites after the WTS were analyzed over time by one-way repeated measures, ANOVA, followed by Tukey’s multiple comparison test when a significant time effect was found to affect the time points that differed from the baseline values.

## 3. Results

**Figure 1:**
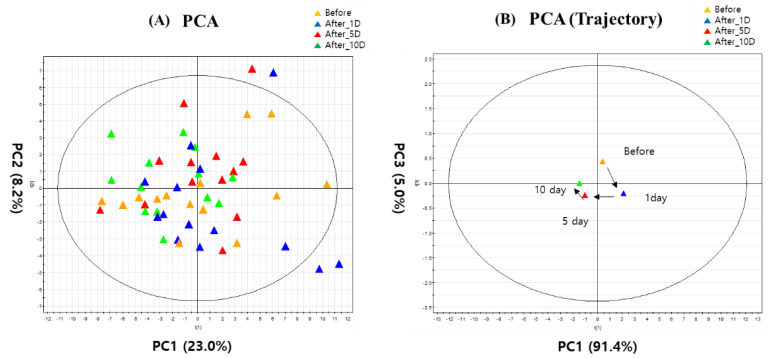
(**A**) PCA and (**B**) trajectory PCA score plots based on identified urinary metabolites. Before: before WTS; After_1D: 1 day after WTS; After_5D: 5 days after WTS; After_10D: 10 days after WTS.

After the NMR analysis of urine samples obtained from young soccer players, 79 metabolites were identified using the Chenomx NMR Suit program (Table S2). For the target profiling of the 79 metabolites, PCA and PCA trajectory score plots are represented in Figure 1. Each subject in the 4 groups is scattered among the PCA plots, whereas the PCA trajectory score plot is shown as divided patterns by each group (Figure 1A). In the PCA trajectory score plot (Figure 1B), based on R2 and Q2 (Table S3), before and 1 day after WTS were separated from 5 and 10 days after WTS by PC1. Although 1 day after WTS was not separated from before WTS, it is necessary to investigate how metabolic change happened after WTS in order to suggest the proper recovery time for young soccer players.

**Figure 2:**
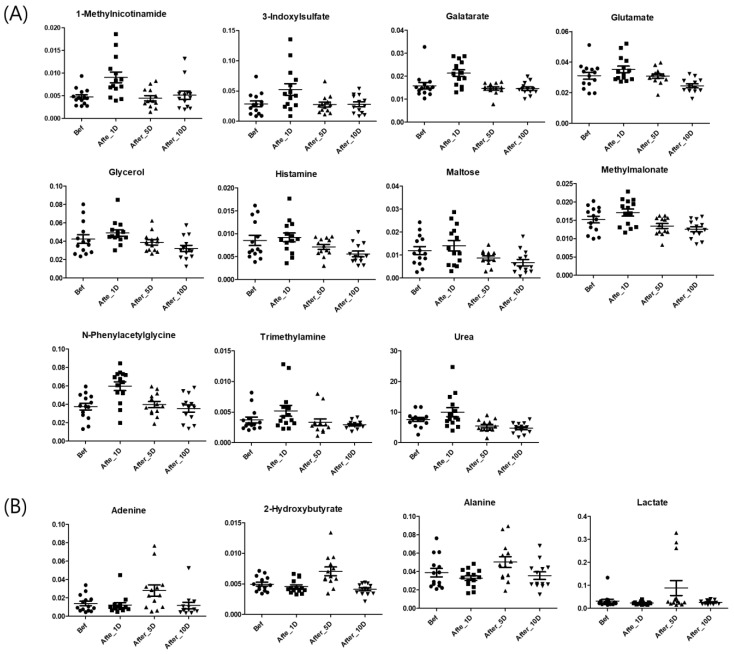
Dot plots of selected metabolites increased 1 day (**A**) and 5 days (**B**) after WTS. Bef: before WTS; Afte_1D: 1 day after WTS; After_5D: 5 days after WTS; After_10D: 10 days after WTS.

PLS-DA between before and 1 (or 5, or 10) days after WTS was conducted (Figure S2). Each of the two groups in the three PLS-DA score plots was divided by PLS 1, and each model validated by the permutation test was well-fitting. Based on the separation, 56 metabolites (VIP > 0.9) were selected among the before and 1 or 5 days after WTS groups. Of these, the following 15 metabolites were selected based on the significance value (*p* < 0.05) obtained during ANOVA (Tukey’s multiple comparison test) (Figure 2): 1-methylnicotinamide, 3-indoxylsulfate, galactarate, glutamate, glycerol, histamine, methylmalonate, maltose, *N*-phenylacetylglycine, trimethylamine, urea, 2-hydroxybutyrate, adenine, alanine, and lactate. Most selected metabolites, except for 2-hydroxybutyrate, adenine, alanine, and lactate, were found to have increased in urine samples 1 day after the WTS. The main metabolic pathways related to the selected metabolites were the urea cycle, nicotinate and nicotinamide, propanoate, histidine, alanine metabolism, malate-aspartate shuttle, and glucose-alanine cycle (Figure S3).

## 4. Discussion

**Figure 3:**
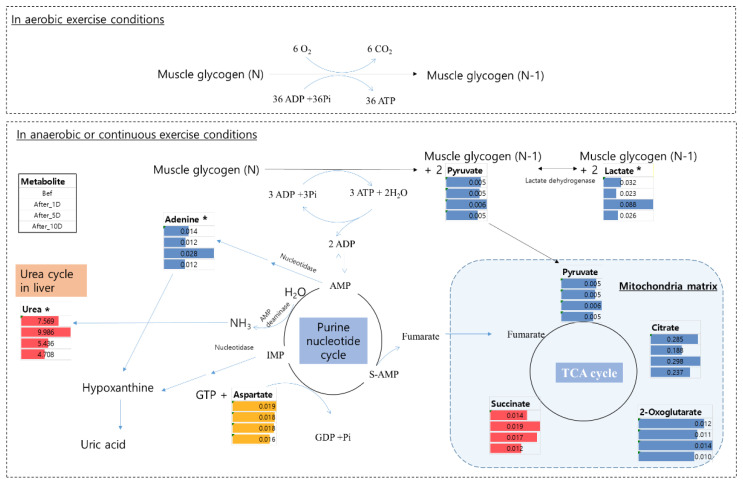
Metabolic change of potential biomarkers in aerobic and anaerobic (or continuous) conditions. * means significantly different metabolite compared to before WTS. Bef: before WTS; After_1D: 1 day after WTS; After_5D: 5 days after WTS; After_10D: 10 days after WTS.

In the WTS in this study, young players exercised for 10 days (Figure S1). During strenuous exercise, adenosine triphosphate (ATP) is produced simultaneously through oxidative and substrate-level phosphorylation in the skeletal muscles [23]. When the ATP runs low or additional energy is needed for exercise, muscle glycogen is degraded to produce ATP. Under aerobic exercise conditions, 36 ATP molecules are obtained from one glucose molecule through oxidative phosphorylation. Although oxidative phosphorylation has a higher capacity than substrate-level phosphorylation, it is inefficient in rapidly producing energy during intense exercise or in the absence of electron donors such as oxygen (O_2_) [23]. Therefore, during anaerobic or continuous exercise conditions, muscle glycogen is degraded, and pyruvate is converted to lactate via substrate-level phosphorylation (Figure 3) [23,24].

A major pathway for the production of ammonia by adenylate deaminase catalyzes the conversion of adenosine 5′-monophosphate (AMP) into inosine 5′-monophosphate (IMP), which is called the purine nucleotide cycle [25] (Figure 3, Equation (1)).

AMP^2−^ + H_2_O → IMP^3−^ + NH_3_ (1)

In this cycle, fumarate is used as the electron acceptor instead of oxygen. Therefore, the overall reaction is as in Equation (2):

Aspartate + GTP + H_2_O ↔ Fumarate + GDP + P_i_ + NH_3_ (2)

Excess ammonia from these metabolic processes is toxic and induces neurotransmitter disturbances, brain swelling, and metabolic disturbances [26]. Therefore, ammonia produced during exercise is metabolically detoxified to produce urea via the urea cycle in the liver and, finally, excreted in the urine. In a study involving 70 km cross-country skiers, urinary urea content increased [27] until the recording ended (1 day after the race). Similar to this study, the level of urea in the urine of young soccer players was significantly increased 1 day after the WTS (Figure 2) and decreased after 10 days of recovery.

**Figure 4:**
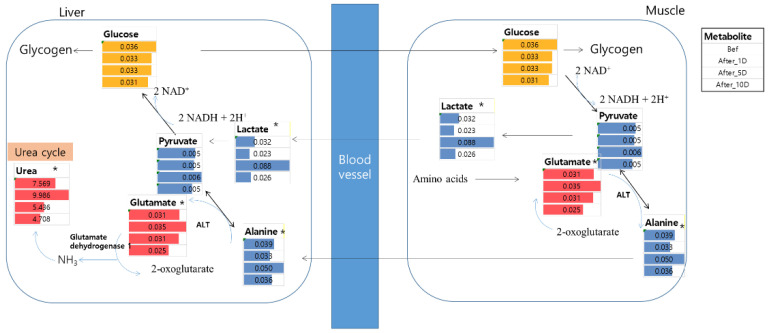
Metabolic change of potential biomarkers in the glucose-alanine cycle. * means significantly different metabolite compared to before WTS. Bef: before WTS; After_1D: 1 day after WTS; After_5D: 5 days after WTS; After_10D: 10 days after WTS.

During exercise, exercise-induced glycogen or glucose depletion occurs, and protein breakdown and amino acid oxidation are also increased to use amino acids as energy sources [28,29]. This is the glucose-alanine cycle (Figure 4), wherein muscle protein is degraded to provide more glucose, which generates additional ATP for muscle contraction. In this pathway, a degraded amino acid containing an amino group, such as glutamate, is converted to 2-oxoglutarate by alanine aminotransferase (ALT) and forms alanine in the muscle. ALT, as an enzyme, also transfers amino groups to pyruvate and 2-oxoglutarate. This alanine flows through the bloodstream and is regenerated to glutamate and pyruvate by ALT in the liver. Then, pyruvate is converted into glucose through gluconeogenesis, and subsequently, glutamate is re-catabolized into 2-oxoglutarate by glutamate dehydrogenase (GDH), which also produces ammonium ions that move to the liver to form urea in the urea cycle. Therefore, the glucose-alanine cycle is not only a way to produce additional ATP during exercise but also to eliminate ammonia from the body to prevent its build-up in the muscles. 

Among the intermediates of the glucose-alanine cycle, glutamate is an excitatory neurotransmitter in the central nervous system (CNS), which affects learning and memory. However, elevated plasma levels of glutamate also induce neuropsychiatric disorders, such as epilepsy and Alzheimer’s disease [30,31,32]. In addition, it is involved in muscle fiber activity. Cairns et al. have reported that intramuscular injection of glutamate (1.0 M) elicits sensitization of rat afferent fiber activity by activating peripheral excitatory amino acid receptors [33]. Thus, it is important to eliminate the excess glutamate from the body. Jang et al. have reported that the level of plasma glutamate at 5 min after exercise increased in horses [34]. In the current study, the glutamate level increased in the urine 1 day after the WTS and decreased up to 10 days after recovery. After the exercise ended, excess ammonia seemed to get converted into glutamate by GDH. GDH converts glutamate to α-ketoglutarate; conversely, it is involved in the amination of α-ketoglutarate to maintain ammonia homeostasis (Figure S3). Wibom et al. have reported that glutamate dehydrogenase levels increased after training or detraining [35]. In addition, GDH is involved in ammonia fixation and the synthesis of glutamate upon the occurrence of hyperammonemia, meaning a high level of ammonia in the blood [36]. Therefore, a high glutamate level seems to be excreted in the urine to regulate ammonia levels in the plasma.

In urinary analysis, metabolites were normalized with creatinine concentrations assuming that all the glomerular functions would be normal. Urinary creatinine level is a biomarker for kidney function. The creatinine concentrations of all urine samples were within 23.7~299.7 mg/dL, implying normal kidney function of the young soccer players based on the urinary creatinine. Although young soccer players did not perform intense or hard exercises during the WTS, exercise might induce acute kidney injury [37,38]. Some urinary metabolites are related to renal dysfunction and are elevated in plasma or urine during renal dysfunction. Increased excretion of trimethylamine has been reported in patients with renal disease, a condition characterized by decreased activity of flavin-containing monooxygenase (EC 1.14.13.8) isoform 3 enzyme (FMO3), which induces the accumulation of trimethylamine in urine [39]. In addition, methylmalonate is related to impaired renal function, and elevated levels of methylmalonate in urine increase the risk of mortality [40]. As mentioned above, protein degradation occurred during exercise, and ketogenic amino acids, such as valine, threonine, isoleucine, and methionine, induced the production of propionyl- and methylmalonyl-CoA, which are pre-metabolites of succinyl-CoA in the TCA cycle [41]. Methylmalonate is a substrate of methylmalonyl-CoA mutase and inhibits succinate dehydrogenase. Therefore, accumulated methylmalonate acts as a neurotoxin in the brain [42]. In addition, phenylacetylglycine is increased in the urine of patients with chronic kidney disease and is metabolized by the gut flora [43].

At substrate-level phosphorylation, lactate is produced as a by-product of lactate dehydrogenase (LDH), which is induced by exercise in human muscles [44]. Lactate produced during exercise acts as an energy source and signaling molecule, but also induces adverse effects in muscles, such as muscle fatigue, pH alteration, and damage to the liver, muscles, or kidneys, as well as leads to the production of ammonia [45,46]. After exercise, to remove lactate, it is rapidly oxidized to CO_2_ (55–70%) and converted to glucose through the glucose-alanine cycle (Figure 4) in the muscles and liver (<20%); hence, an elevated level of lactate during exercise was usually observed to have decreased within 1 h after recovery [47]. This is important for preventing cell damage upon lactate accumulation, which induces muscle fatigue, muscle fiber damage, and inflammation. In our study, lactate levels 1 day after the WTS did not differ from those before exercise. In addition, the level of another intermediate in the glucose-alanine cycle, viz. alanine, was also not altered 1 day after exercise. As mentioned above, alanine in the muscles is converted from glutamate and pyruvate through ALT activity in the middle of the exercise [48]. According to a study by Clifford et al., ALT and LDH levels increased immediately after exercise [49]. Therefore, no differences in lactate level were observed 1 day after exercise as compared to before exercise. In patients with lactic acidosis and ketoacidosis, the level of 2-hydroxybutyrate increases with an increase in lactate excretion after physical exercise [50]. Even in non-patients (healthy individuals), lactic acidosis is caused by the accumulation of pyruvate converted to lactate under hypoxic conditions. This is caused by an increase in the NADH_2_/NAD ratio in the cytoplasm, which stems from cellular hypoxia [50]. Alanine and lactate levels in the urine have been reported in many studies to increase during exercise and return to normal values within 24 h [51]. However, the levels of lactate and alanine at 5 days after WTS were found to have increased (Figure 2) in this study. Some enzymes remain at high levels after exercise, and there are many differences between individuals. Pettersson et al. have reported that lactate dehydrogenase, ALT, and other liver enzymes increased after weightlifting exercise for 4 or 5 days [52].

Adenine is a purine compound that takes part in the formation of DNA and RNA by getting attached to deoxyribose (d-adenosine) and ribose (adenosine), respectively. AMP, adenosine diphosphate (ADP), and ATP are d-adenosine phosphates (adenine nucleotides) involved in energy storage and transfer. During exercise, some adenine nucleotides (AMP) are degraded by AMP deaminase to IMP, and ammonia in the muscles is released to the liver for detoxification [25,53]. It is also well known that intense exercise induces the degradation of adenine nucleotides and increases adenosine and adenine levels. When considering purine metabolism, intense exercise induces high adenine concentration in the plasma, which lasts for 3 h post-exercise [54]; the concentration then quickly returns to a normal level. Moreover, adenine has been shown to be an acute kidney injury-induced compound in many studies [55]. A diet containing adenine contributes to increased levels of serum uric acid and induces other markers that cause renal disorders [56].

In this study, most selected metabolites increased 1 day after the WTS and then returned to normal levels. However, 4 metabolites, namely adenine, 2-hydroxybutyrate, alanine, and lactate, increased during 5 days of the recovery period after the WTS. In particular, because adenine and lactate are strong biomarkers of renal toxicity and fatigue, respectively, more than 5 days of recovery after the WTS might be appropriate for the health of young soccer players.

## 5. Conclusions

Urinary metabolites in young players before and 1, 5, and 10 days after the WTS were analyzed by NMR combined with multivariate analysis to suggest appropriate recovery times. In the NMR analysis, 79 metabolites were identified, and each group was clustered using PLS-DA. The selected metabolites contributing to these clusters were 1-methylnicotinamide, 3-indoxylsulfate, galactarate, glutamate, glycerol, histamine, methylmalonate, maltose, *N*-phenylacetylglycine, trimethylamine, urea, 2-hydroxybutyrate, adenine, alanine, and lactate, and were mainly involved in the urea, purine nucleotide, and glucose-alanine cycles. In this study, most selected metabolites were increased 1 day after the WTS, except for 4 metabolites, namely adenine, 2-hydroxybutyrate, alanine, and lactate. Based on excess ammonia, adenine, and lactate levels in the urine, an appropriate recovery time was determined to be at least 5 days.

## Data Availability

The original contributions presented in the study are included in the article; further inquiries can be directed to the corresponding authors.

## Supplementary Materials

The following supporting information can be downloaded at: https://www.mdpi.com/article/10.3390/metabo12121283/s1. Figure S1: Schematic diagram of the experimental schedule in the study; Figure S2: PLS-DA plots, parameters, and results of permutation test between before and 1 (A) (or 5 (B), or 10 (C)) day after WTS; Figure S3: Metabolisms related to selected biomarkers (A) between before and 1 day after WTS and (B) between before and 5 days after WTS; Figure S4: Overall pathway related to selected biomarkers; Table S1: Physical properties including height, body weight, muscle, body fat, and body mass index (BMI) of the individual subjects; Table S2: Fold-change and VIP of identified metabolites between before and 1, 5 or 10 days after WTS; Table S3: Parameters of multivariate analysis in PCA and PCA trajectory score plot in urine.
