# Supplementary material for: Urinary Metabolomics in Young Soccer Players after Winter Training Season
Source: Metabolites. 2022 Dec 17;12(12):1283. doi: 10.3390/metabo12121283 (PMC9784126; doi:10.3390/metabo12121283)

Table S1. Physical properties including height, body weight, muscle, body fat, and body mass index (BMI) of the individual subjects

	Height (cm)	Body weight (kg)	Muscle mass (kg)	Body mass index (BMI)	Body fat (kg)
Subject1	145.4	40.3	18.8	19.1	14.3
Subject2	144.1	42.5	17.9	20.5	20.8
Subject3	143.2	36.1	16.6	17.6	13.0
Subject4	143.7	32.3	14.7	15.6	12.4
Subject5	141.8	41.9	16.2	20.8	26.6
Subject6	153.8	39.9	19.3	16.9	10.0
Subject7	148.4	33.1	15.9	15.0	8.9
Subject8	142.0	29.8	13.8	14.8	10.4
Subject9	145.3	32.0	14.3	15.2	13.4
Subject10	140.1	32.9	14.6	16.8	15.2
Subject11	136.2	34.1	14.2	18.4	19.1
Subject12	141.8	35.4	14.8	17.6	19.2
Subject13	141.1	40.8	16.5	20.5	24.0
Subject14	137.5	31.0	14.2	16.5	13.0
Mean	143.2	35.9	15.8	17.5	15.7
SD	4.4	4.4	1.8	2.1	5.4

Figure S1. Schematic diagram of the experimental schedule in the study.

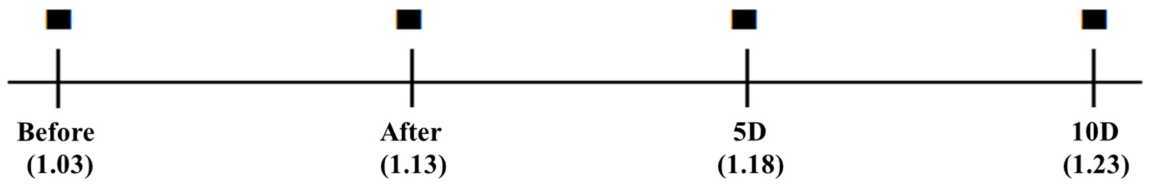

<Morning training>

Exercise		Duration	Day
Technical drills	Coordination, Ball control, 5 : 2 passing, passing game, dribbling, shooting, crossing, ball heading,	25 min	Mon, Wed, Fri,
Tactical drills	Set piece, strategies (Attacking, defending, corner kicks)	25 min	
Mini games		40 min	
Friendly match		90 min	Tue, Thu

<Afternoon training>

Complex physical training program

Order	Exercise type	Time	Intensity	Day
Warm-up	Stretching	5 min		
FIFA 11+ Kids	Running game	20 min	5 stop / 3 set	Mon, Wed, Fri
	Skating jumps		6 each / 3set	
	Single leg jumps		5 per leg / 3 set	
	Ball passing		5 per leg / 3 set	
	Spider man		3 set	
	Push-up		20 sec / 3 set	
	Falling techniques		5-7 per side / 1 set	
Resistance training	Lunge	30 min	12-20 reps / 3 sets, Rest between set: 30 sec, Rest between exercise: 1 min	
	Single-leg calf raise			
	Back extension			
	Bicycle crunch			
Coll-down	Stretching	5 min		

Table S2. Fold-change and VIP of identified metabolites between before and 1, 5 or 10 day after WTS in PLS-DA.

No.	Metabolites	Fold change / VIP					
		After_1D/Before		After_5D/Before		After_10D/Before	
		Fold change	VIP[1]	Fold change	VIP[1]	Fold change	VIP[1]
1	1,3-Dihydroxyacetone	1.10	0.46	0.90	0.54	0.96	0.20
2	1,3-Dimethylurate	1.00	0.00	0.71	**1.42**	0.66	**1.63**
3	1-Methylnicotinamide	1.91	**2.39**	0.94	0.33	1.08	0.31
4	2-Aminoadipate	0.98	0.18	0.88	**0.98**	0.78	**1.60**
5	2-Hydroxy-3-methylvalerate	0.92	**0.92**	1.04	0.55	1.00	0.03
6	2-Hydroxybutyrate	0.92	0.75	1.42	**2.15**	0.83	**1.58**
7	2-Hydroxyisobutyrate	0.95	0.45	0.90	**0.96**	0.92	0.70
8	2-Hydroxyphenylacetate	1.37	0.88	0.93	0.31	0.78	**1.67**
9	2-Octenoate	1.33	**1.01**	0.99	0.03	1.01	0.04
10	2-Oxoglutarate	0.89	0.70	1.12	0.64	0.85	**0.92**
11	2-Oxoisocaproate	0.84	**1.78**	0.93	0.80	0.89	1.36
12	2-Phenylpropionate	0.87	**0.95**	0.82	**1.38**	0.95	0.31
13	3-Hydroxybutyrate	1.03	0.31	0.90	**0.98**	0.88	1.03
14	3-Hydroxyisovalerate	1.10	0.70	1.12	0.76	1.18	**1.18**
15	3-Indoxylsulfate	1.84	**1.74**	0.97	0.13	0.98	0.09
16	3-Methyl-2-oxovalerate	1.03	0.41	0.99	0.10	0.94	0.88
17	3-Phenyllactate	1.22	**1.07**	0.86	0.66	0.78	**1.24**
18	4-Aminobutyrate	1.10	0.71	1.07	0.72	1.00	0.01

19	Acetate	1.14	0.60	1.16	**0.92**	0.91	0.49
20	Acetoacetate	1.20	**1.00**	0.86	**0.96**	0.89	0.72
21	Acetone	1.14	**1.13**	1.25	**1.57**	1.02	0.19
22	Adenine	0.88	0.40	2.02	**1.81**	0.84	0.43
23	Alanine	0.85	**0.93**	1.30	**1.35**	0.92	0.43
24	Anserine	0.78	**1.00**	0.80	**0.90**	0.52	**2.04**
25	Arginine	1.06	0.32	1.04	0.19	1.04	0.18
26	Asparagine	0.89	0.69	1.06	0.35	0.93	0.46
27	Aspartate	0.97	0.24	0.95	0.46	0.86	**1.21**
28	Betaine	0.79	**1.21**	0.76	**1.31**	0.58	**2.07**
29	Caffeine	0.93	0.36	0.82	**0.91**	0.69	**1.35**
30	Carnitine	1.18	0.60	1.49	**1.54**	0.94	0.17
31	Choline	0.89	0.74	1.05	0.32	0.87	**1.06**
32	Citrate	0.66	**1.67**	1.04	0.20	0.83	0.79
33	Creatine	1.30	0.68	1.47	**1.00**	2.34	**1.57**
34	Creatine phosphate	1.20	**0.93**	1.05	0.23	0.93	0.34
35	Dimethyl sulfone	0.73	**1.70**	0.66	**2.54**	1.02	0.09
36	Dimethylamine	1.00	0.01	0.99	0.09	1.03	0.23
37	Formate	1.01	0.05	0.84	0.75	0.98	0.09
38	Galactarate	1.35	**2.04**	0.93	0.64	0.92	0.63
39	Galactose	0.74	0.68	0.79	0.59	0.78	0.58
40	Glucose	0.92	0.71	0.91	**1.06**	0.85	**1.43**
41	Glutamate	1.13	**1.09**	0.99	0.08	0.79	**1.88**

42	Glutamine	1.13	**1.20**	1.09	0.82	1.08	0.78
43	Glutarate	1.12	**0.92**	1.15	0.73	1.05	0.33
44	Glutathione	0.81	**1.18**	0.79	**1.50**	0.64	**2.23**
45	Glycerol	1.16	**0.95**	0.92	0.56	0.76	**1.38**
46	Glycine	0.62	**1.87**	0.85	0.70	0.94	0.30
47	Glycolate	0.82	0.58	1.00	0.00	0.74	0.84
48	Hippurate	1.19	0.55	1.71	0.87	1.24	0.49
49	Histamine	1.08	0.38	0.83	**0.98**	0.66	**1.73**
50	Histidine	0.98	0.08	1.18	0.68	0.90	0.54
51	Isobutyrate	1.04	0.25	1.40	**1.35**	1.05	0.32
52	Lactate	0.72	**0.91**	2.78	**1.48**	0.81	0.58
53	Lactose	0.81	0.71	0.75	**0.95**	0.84	0.55
54	Leucine	1.19	**1.69**	1.19	**1.84**	1.00	0.02
55	Malonate	1.06	0.47	0.96	0.33	0.96	0.37
56	Maltose	1.18	0.64	0.73	**1.36**	0.56	**1.76**
57	Mannitol	0.69	**1.09**	0.83	0.56	0.93	0.16
58	Methionine	1.07	0.43	1.38	**1.43**	1.00	0.01
59	Methylamine	1.13	**0.92**	1.02	0.13	0.96	0.33
60	Methylguanidine	0.80	**1.02**	1.21	**0.90**	1.05	0.19
61	Methylmalonate	1.12	**1.16**	0.88	**1.32**	0.83	**1.73**
62	N,N-Dimethylglycine	1.02	0.10	0.92	0.40	0.93	0.33
63	N-Acetylglucosamine	0.87	0.63	0.76	**1.25**	0.77	**1.08**
64	N-Acetyltyrosine	1.22	**1.84**	1.02	0.17	0.99	0.08

65	N-Phenylacetylglycine	1.59	**2.53**	1.06	0.39	0.94	0.14
66	O-Acetylcholine	1.16	0.61	1.29	**1.12**	1.02	0.08
67	Pyruvate	0.97	0.20	1.16	0.59	0.92	0.45
68	Succinate	1.35	**1.43**	1.27	**0.99**	0.85	0.63
69	Succinylacetone	1.08	0.63	0.92	0.84	0.90	**0.90**
70	Taurine	0.95	0.30	1.26	**1.30**	1.06	0.29
71	Thymol	0.94	0.49	0.77	**1.51**	0.75	**1.62**
72	Trigonelline	1.00	0.01	1.07	0.21	0.92	0.25
73	Trimethylamine	1.41	**1.20**	0.89	0.49	0.79	**1.20**
74	Trimethylamine N-oxide	0.48	**1.40**	1.26	0.47	1.51	0.70
75	Urea	1.32	**1.22**	0.72	**2.01**	0.62	**2.42**
76	Uridine	1.41	0.91	1.21	**1.38**	0.93	0.69
77	Valine	0.98	0.19	1.07	0.74	1.13	0.98
78	cis-Aconitate	0.98	0.18	1.13	**1.10**	0.89	0.89
79	trans-Aconitate	1.01	0.03	0.97	0.25	0.85	0.95

Table S3. Parameters of multivariate analysis in PCA and PCA trajectory score plot in urine.

PCA	R2X	R2X(cum)	Eigenvalue	Q2	Limit	Q2(cum)	Significance
1	0.23	0.23	12.4	0.158	0.0301	0.158	R1
2	0.0824	0.313	4.45	-0.0123	0.0305	0.148	R2

PCA trajectory	R2X	R2X(cum)	Eigenvalue	Q2	Limit	Q2(cum)	Significance
1	0.914	0.914	3.66	0.401	0.259	0.401	R1
2	0.0504	0.965	0.202	-0.116	0.341	0.341	N3
3	0.0352	1	0.141	0.999	0.506	1	N3

Figure S2. PLS-DA plots, parameters and results of permutation test between before and 1 (A) (or 5 (B), or 10(C)) day after WTS.

(A)

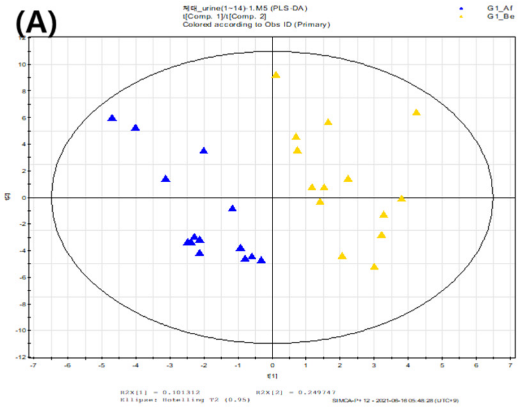

(B)

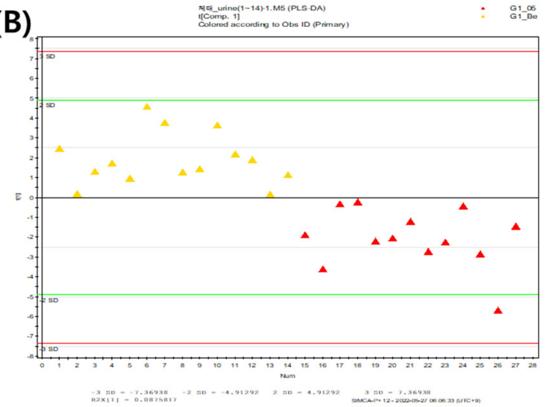

(C)

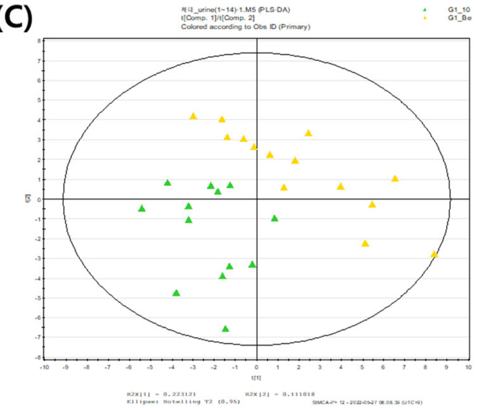

A	R2X	R2X(cum)	Eigenvalue	R2Y	R2Y(cum)	Q2	Limit	Q2(cum)	Significance
0	Cent.			Cent.					
1	0.101	0.101	2.84	0.743	0.743	0.45	0.05	0.45	R1
2	0.25	0.351	6.99	0.107	0.851	0.212	0.05	0.567	R1
3	0.0564	0.407	1.58	0.0973	0.948	0.274	0.05	0.686	R1

A	R2X	R2X(cum)	Eigenvalue	R2Y	R2Y(cum)	Q2	Limit	Q2(cum)	Significance
0	Cent.			Cent.					
1	0.0876	0.0876	2.36	0.685	0.685	0.0735	0.05	0.0735	R1

A	R2X	R2X(cum)	Eigenvalue	R2Y	R2Y(cum)	Q2	Limit	Q2(cum)	Significance
0	Cent.			Cent.					
1	0.223	0.223	6.02	0.4	0.4	0.192	0.05	0.192	R1
2	0.111	0.334	3	0.352	0.752	0.369	0.05	0.49	R1

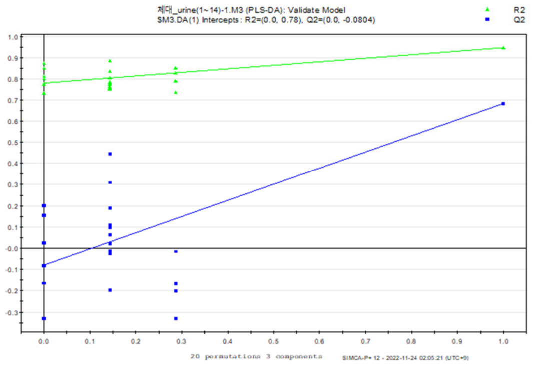

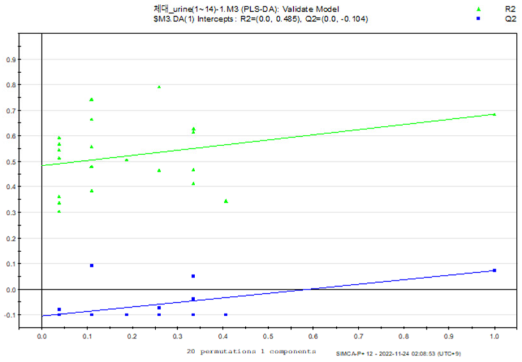

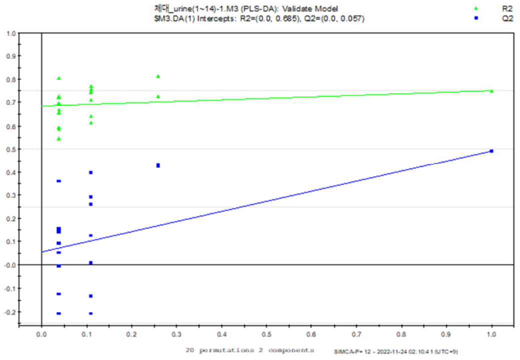

Figure S3. Metabolisms related to selected biomarkers (A) between before and 1 day after WTS and (B) between before and 5 days after WTS.

(A)

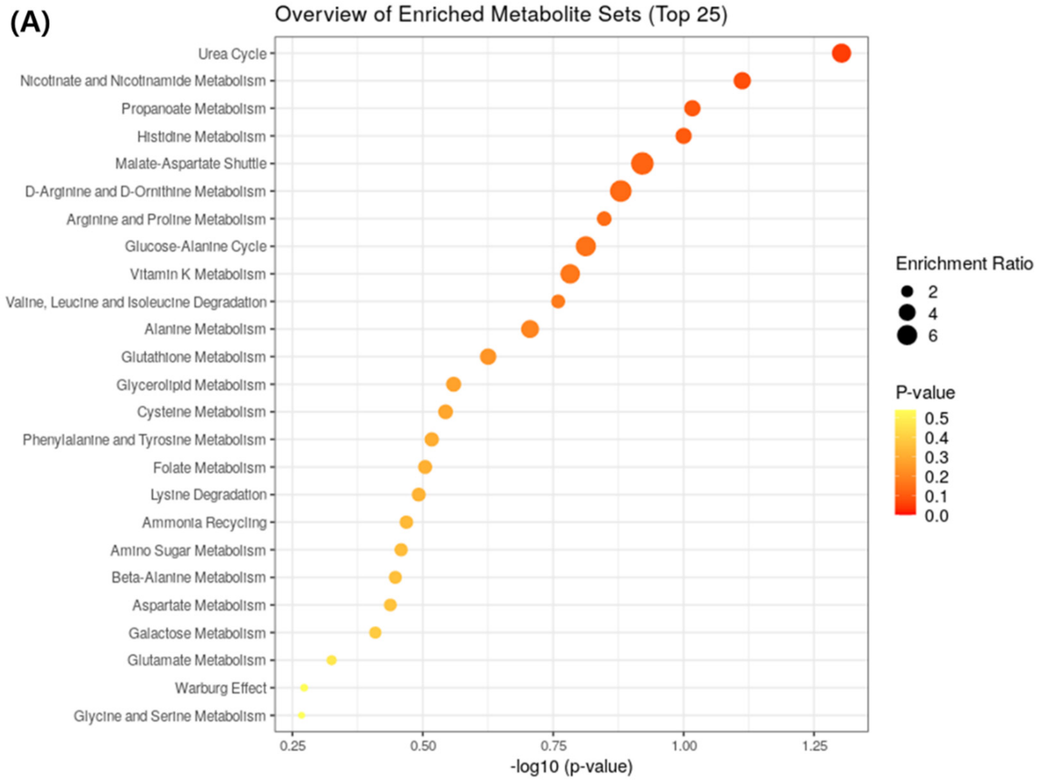

(B)

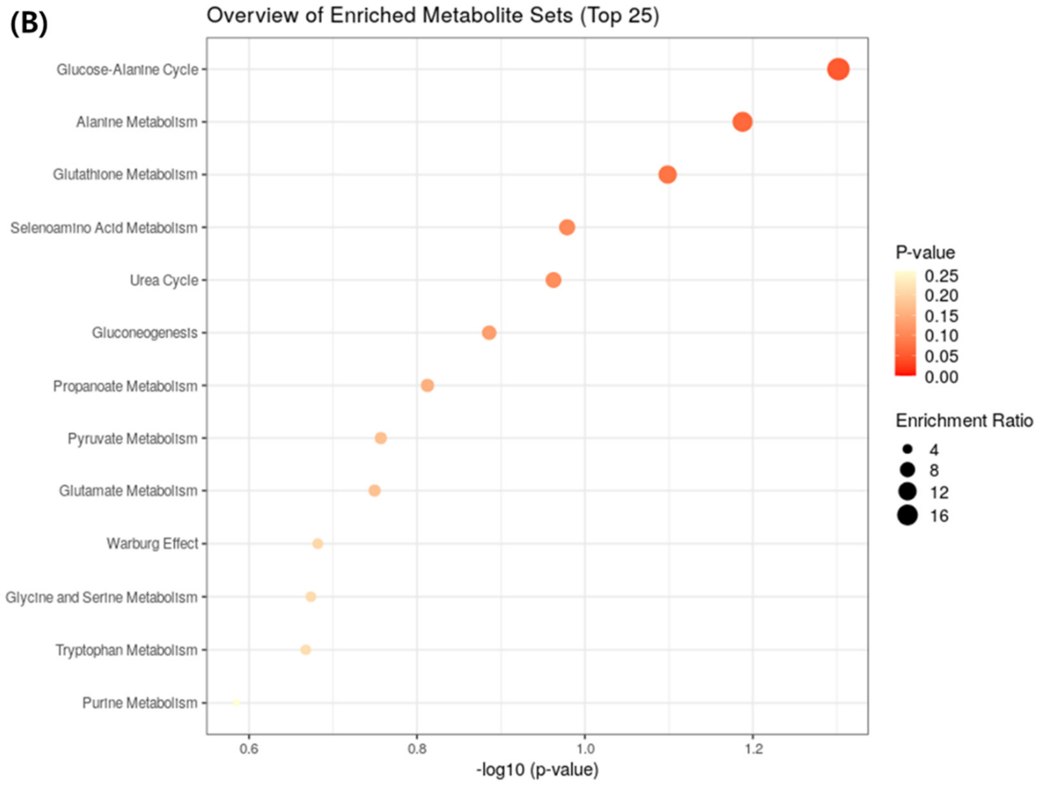

Figure S4. Overall pathway related to selected biomarkers.

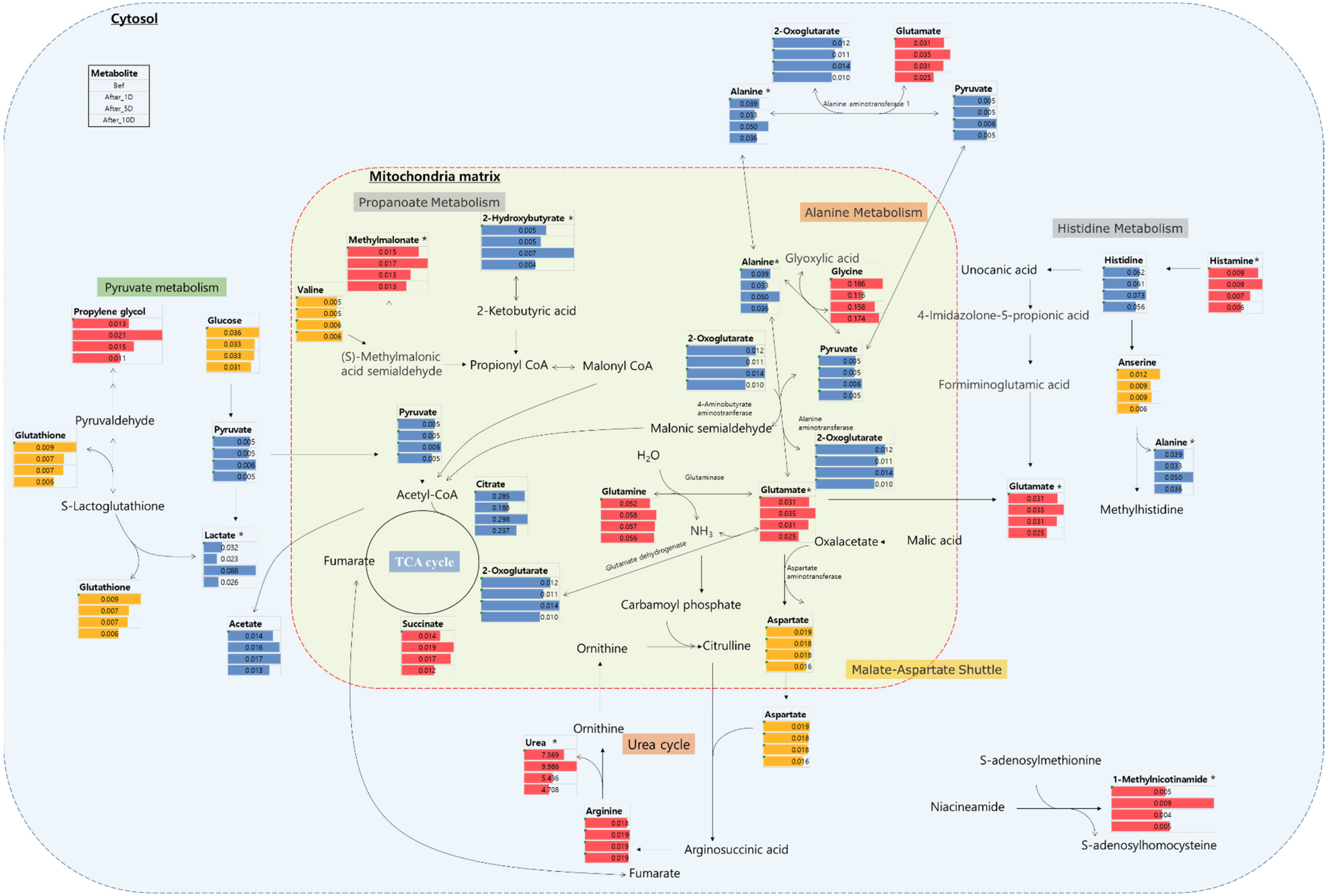

Supplement: Supplementary file 1 [file metabolites-12-01283-s001.zip › metabolites-2050900-supplementary.pdf]
